# Prevalence of HIV/AIDS among pregnant women in North American region: A systematic review and meta-analysis

**DOI:** 10.1097/MD.0000000000040339

**Published:** 2024-11-01

**Authors:** Nosheena Akhter Shabbir, Sara Bashir Kant, Kainat Rashid, Uzma Hafeez, Aiza Ali Akbar, Syeda Wajiha Batool, Alif Hasan Pranto, Jemema Zaman, Hasan Shahriyer Tonmoy, Md Rashedul Islam, M. M. Rubaiyat Muntasir Meem, Dewan Zubaer Islam, Ehsan Suez, Shahad Saif Khandker, Amna Akbar, Muhammad Iftikhar Khattak, Amir Iqbal Ali, Sarosh Khan Jadoon, Attarab Shakeel, Maryam Zubair, Sarosh Alvi

**Affiliations:** a Department Obstetric and Gynecology, Azad Jammu & Kashmir Medical College, Muzaffarabad, Pakistan; b Azad Jammu & Kashmir Medical College, Muzaffarabad, Pakistan; c Resident Emergency Medicine, Combined Military Hospital Rawalpindi, Rawalpindi, Pakistan; d Department of Community Medicine, Azad Jammu & Kashmir Medical College, Muzaffarabad, Pakistan; e School of Pharmacy, Brac University, Dhaka, Bangladesh; f Department of Health Policy and Management, University of Georgia, Athens, GA; g Department of Microbiology, Jahangirnagar University, Dhaka, Bangladesh; h Institute of Bioinformatics, University of Georgia, Athens, GA; i Department of Microbiology, Gonoshasthaya Samaj Vittik Medical College, Dhaka, Bangladesh; j Medical Officer District Headquarter Hospital Hattian, Muzaffarabad, AJK, Pakistan; k Celestial & Dimanche, Muzaffarabad, AJK, Pakistan; l Combined Military Hospital/SKBZ, Muzaffarabad, AJK, Pakistan; m Azad Jammu & Kashmir Medical College, Classifies Gynecologist, Combined Military Hospital/SKBZ, Muzaffarabad, AJK, Pakistan; n University of Khartoum, Khartoum, Sudan.

**Keywords:** AIDS, epidemiology, frequency, HIV, maternal, pregnancy

## Abstract

**Background:**

As a major maternal health concern, the prevalence of human immunodeficiency virus (HIV) among pregnant women was previously investigated in European, African, and Latin American regions other than the North American region. This study analyzed the prevalence of HIV among pregnant women in the North American region including 3 major countries: the USA, Canada, and Mexico.

**Methods:**

Relevant studies were screened from 3 online databases: Google Scholar, PubMed, and ScienceDirect using specific search keywords. Ultimately, 10 studies of the North American region were included with a total of 339,831 pregnant women residing in the USA, Canada, and Mexico.

**Results:**

The overall pooled prevalence was 0.6% (95% confidence interval [CI]: 0.4–0.8) with a high degree of heterogeneity (*I*^2^ = 97%). Pooled prevalence rates of HIV among pregnant women in Canada, Mexico, and the USA were 0.3% (95% CI: 0.1–0.5), 0.5% (95% CI: 0.2–0.8), and 2.3% (95% CI: 0.0–5.7), respectively with high degrees of heterogeneity.

**Conclusion:**

The overall prevalence rate of HIV among pregnant women in the USA, Canada, and Mexico was minimal as compared with the countries of Eastern Europe, sub-Saharan Africa, or Latin America. Awareness, adequate testing and healthcare facilities, better socioeconomic, and geopolitical conditions might be crucial to lowering the prevalence of HIV among pregnant women.

**Key points**

• The prevalence of HIV/AIDS among pregnant women within the North American region was determined as 0.6% (95% CI: 0.4–0.8).
• In Canada and Mexico, the rate was found below 1%.
• In the USA, the rate was determined 2.3% (95% CI: 0.0–5.7).
• Adequate testing, public awareness, and following healthcare guidelines can reduce the burden of HIV among pregnant women to zero.

## 1. Introduction

The human immunodeficiency virus (HIV), which is the ultimate cause of acquired immunodeficiency syndrome (AIDS) remains a major global public health concern, with millions of lives lost and ongoing transmission in all countries.^[1–3]^ According to the WHO, there have been approximately 40.4 million deaths due to continuous worldwide transmission while around a total of 39 million people including 0.7% of adults aged 15 to 49 are living with HIV worldwide as per data till 2022. WHO reports suggest that the number of people acquiring HIV has been reduced by 38% in 12 years (2010–2022), approximately from 2.1 million to 1.3 million people. It can be assumed that a growing number of people are being aware of the transmission of HIV day by day and taking preventive measures.^[4–6]^ HIV is one of the few diseases that does not come with the natural nemesis of what we call a permanent treatment plan.^[6,7]^ In other words, it cannot be cured but rather it can be kept under control with passive medication to prevent the matter from being worse. That is why there are a number of preventive and long-term palliative treatment measures specifically designed to ail the HIV patients all over the world until permanent HIV treatment is discovered.^[8,9]^ HIV may be transmitted by a variety of bodily fluids, including blood, breast milk, semen, and vaginal secretions of HIV infected persons. Additionally, transmission from a pregnant person to their offspring is possible during pregnancy and childbirth, which is one of the major routes of HIV transmission.^[10,11]^

It is vital to remember that sharing personal belongings, food, or drink as well as common daily encounters like kissing, hugging, and handshakes do not spread HIV.^[4,12,13]^ HIV is an RNA virus belonging to the genus *Lentivirus* within the family of *Retroviridae*.^[14]^ As the body’s immune system attempts to fight off the infection after an infection, a person may have flu-like symptoms. The symptoms disappear within a few weeks; thus an infected individual may remain healthy for decades before any significant signs and symptoms are visible. Afterward, the situation only worsens with time and the person becomes more prone to opportunistic infections. This is due to HIV being active and eliminating the body’s T cells, also known as white blood cells, which are vital for the immunological system to defend our body against foreign and opportunistic pathogens. The body is consequently rendered defenseless because of failing to protect against pathogens or anything that might be harmful or contagious. At this stage, untreated HIV can result in AIDS.^[15,16]^ Untreated infections pose serious problems for clinical care as well as public health. People who are not aware of their infections may unintentionally spread illnesses to their sexual partners, the unborn child in the case of pregnant women, and even healthcare workers.^[17]^

HIV-infected women who get pregnant or pregnant women who get infected with HIV are at high risk of maternal and perinatal morbidity and mortality along with the risks of vertical transmission to the fetus during pregnancy labor, delivery, and breastfeeding.^[18,19]^ According to various sources, there are still many reports of maternal HIV conditions which contribute to the number of transmissions worldwide. Although it is quite devious work to reduce the number of infected children, there are a few options and therapy designed for this kind of situation. In 2022, there were approximately 1.2 million pregnant women living with HIV worldwide; 82% (64–98%) of these women got antiretroviral therapy to reduce the risk of transmission from mother to child. Each year, an estimated 1.3 million women and girls who are HIV-positive experience pregnancy.^[20,21]^ There are several key risk factors and complications related to HIV positive pregnant women. The common ones are vertical transmission, weakened immune system, susceptibility to opportunistic infections etc. Moreover, HIV is also associated with preterm birth and low birth weight. HIV-positive women may have a higher risk of certain obstetric complications such as preeclampsia and gestational diabetes, which can affect the course of the pregnancy.^[22,23]^ Antiretroviral therapy (ART) for HIV, usually referred to as HIV treatment, is a medical strategy for controlling HIV infection. The basic objectives of ART are to maintain or enhance immune system function, decrease viral load (the quantity of virus in the blood), and inhibit the reproduction of the HIV virus in the body. However, antiretroviral treatment affects pregnant women who are HIV-positive. Antiretroviral medication causes complications and raises the rate of HIV transmission from a mother to her unborn child. Antenatal antiretroviral medication in combination decreased the risk of HIV transmission early in pregnancy.^[24]^

In this systematic review and meta-analysis, the prevalence of HIV infection among pregnant women in 3 major countries i.e., the United States of America (USA), Canada and Mexico of the North American continent was described and analyzed to determine the pooled prevalence. Thus far, no meta-analysis was published in the North American region associated with HIV prevalence among pregnant women.

## 2. Materials and methods

### 2.1. Eligibility criteria

As the objective of this systematic review and meta-analysis was directed toward the prevalence of HIV among pregnant women in North American countries, only the original articles that are relevant to the prevalence, incidence, occurrence, or frequency of HIV among pregnant women residing in North American countries mainly focusing the USA, Canada, and Mexico were considered as eligible. Following the PRISMA “Preferred Reporting Items for Systematic Reviews and Meta-Analyses” method the article was designed. The search contents other than original articles, that is, review articles, systematic review articles, meta-analyses, book chapters, conference info, case reports, correspondence, news, short communications, mini-reviews, editorials, press releases, blogs, data from websites, discussions, abstracts, etc were considered as ineligible for this study and thus not included. Articles that were not written in English language were excluded as ineligible.

### 2.2. Literature search strategies

Three different electronic databases, Google Scholar, PubMed, and ScienceDirect were screened separately using different keywords including “Human immunodeficiency virus,” “HIV,” “AIDS,” “prevalence,” “occurrence,” “incidence,” “frequency,” “epidemiology,” “pregnant,” “maternal,” “prenatal,” “antenatal,” “perinatal” along with the names of North American countries, for example, “USA,” “Canada,” “Mexico” and adjusted with appropriate Boolean operators during searches. “Title and abstract” and “Title, abstract or author-specified keywords” filters were applied in advanced search options during searches in PubMed and ScienceDirect, respectively. Additional filters such as “Full text” and “Research articles” were also used in PubMed and ScienceDirect, respectively. During the searches in Google Scholar the term “allintitle” was used prior to the specific search keywords. The detailed search strategy was added to Table S1, Supplemental Digital Content, http://links.lww.com/MD/N835. The reference list of each included article was also screened carefully for eligible articles. Studies were checked for duplication using EndNote software and excluded from the eligible study list. Year range or publication date filters were not applied in any database during the searches.

### 2.3. Quality assessment and publication bias analyses

Quality as well as validity of all the included studies were evaluated and justified by asking a number of questions acquired from the Study Quality Assessment Tools, National Institute of Health, and Systematic Reviews: Step 6: Assess Quality of Included Studies, University of North Carolina.^[25,26]^ Nine different questions were selected for quality assessment in this study. The possible answers to the questions could be Yes, No, Unclear, NR (not reported), or NA (not applicable) which were converted into numerical numbers, that is, 1 for the answer Yes, 0 for No and Unclear answers, and no score for NR and NA. In the case of NR or NA answer, that question was considered invalid for that study and thus excluded from the score calculation. For each individual study, the overall score was divided by the total number of questions, that is, 9 in this study, and then converted into a percentage which represents the quality along with the risk of bias of that individual study. If the total score of any individual study is ≤50%, then that study was classified as a low-scoring study and thus had a high risk of bias. On the contrary, if any study obtained a total score of ≥80%, then that study was designated as a high-scoring study and thus had a low risk of bias whereas the studies scoring 60% to 70% were considered as moderate-scoring and moderate risk of bias studies based on previous studies.^[27,28]^ Publication bias and subsequent asymmetry were also determined and visually inspected by constructing a funnel plot with Egger test followed by a Galbraith plot using RStudio software (version 4.3.0) and the “metafor” package (version 4.2-0) of R. Studies with high risk of publication bias were visually identified as outlier studies using these 2 plots successively.

### 2.4. Data extraction and analysis

Various types of major characteristic data were extracted from the included studies, that is, study ID, location, study type, duration of the study and places of study setting, participants’ demographics, HIV detection test, HIV test kit/method, and HIV confirmation test kit/method. Study ID included the last name of the first author and the publication year of the respective study whereas the participants’ demographics data section contained data of the study participants, that is, age, number of pregnant women (total), number of HIV infected pregnant women (events) which were extracted carefully to be used in meta-analysis.

A random-effects model with 95% confidence intervals (CIs) method was used to analyze the prevalence of HIV among the pregnant women from the event and total numbers of study participants. *I*^2^ statistics were preferred and used by the authors to determine the heterogeneity of the included studies for meta-analysis. If the value of *I*^2^ was close to zero, homogeneity of the included studies was indicated whereas different ranges of *I*^2^ value indicated different heterogeneity levels of the studies, that is, *I*^2^ value ranging from 25% to 50% indicated low heterogeneity, 51% to 75% indicated moderate heterogeneity and more than 75% indicated substantial heterogeneity.^[29]^ Authors preferred RStudio software (version 4.3.0) and the “metafor” package (version 4.2-0) of R for conducting meta-analysis.

### 2.5. Subgroup analysis

For subgroup analysis to determine the prevalence rate of HIV in pregnant women separately in the USA, Canada, and Mexico, a random effect model was used along with the 95% CI investigation such as the primary analysis. The method of determining the heterogeneity was *I*^2^ statistics and the categorization of the heterogeneity was the same as the method of determining the heterogeneity in the main analysis.^[29]^

### 2.6. Sensitivity analysis

Sensitivity analysis of the included studies was performed by the authors to find out the potential source of heterogeneity and to determine the impact of heterogeneity of the outlier studies on the overall effect size. Firstly, the outlier studies identified in the funnel plot and Galbraith plot with a high risk of heterogeneity were excluded individually and the analysis was repeated in the random-effects model. Secondly, the analysis was conducted using a fixed-effects model instead of a random-effects model.

### 2.7. Patient and public participation and data collection

This systematic review article and meta-analysis data were collected from the included studies. Patients, the public, hospitals, care units, institutions, or third parties were not involved in data collection, study design, assessment, analysis, and interpretation of the results. In case of discrepancies or lack of clarification, only the corresponding author or the first author of the respective study was contacted.

### 2.8. Outcome of the study

These analyses would identify the prevalence of HIV/AIDS among pregnant women within the North American region as well as the prevalence rate in the USA, Canada, and Mexico separately. The findings will help us to decide what steps and guidelines we will require to resolve this problem.

## 3. Results

### 3.1. Search results and study selection

**Figure 1.**
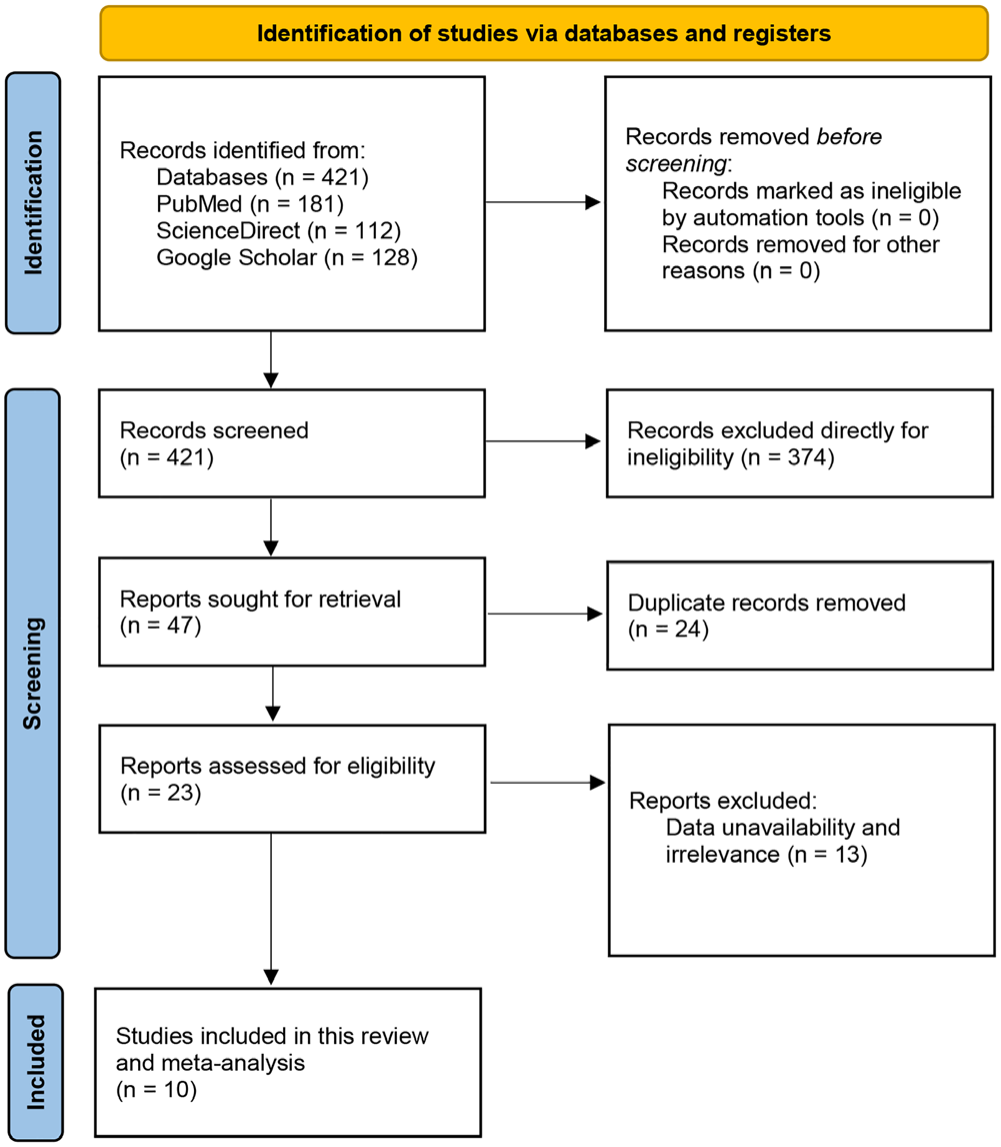
PRISMA flow diagram. PRISMA = the preferred reporting items for systematic reviews and meta-analyses.

Based on our search strategies, keyword combinations and search filters applied in 3 electronic databases, that is, Google Scholar, ScienceDirect, and PubMed a total of 421 search results were found (Google Scholar: 128, ScienceDirect: 112, and PubMed: 181). As only the original articles were required, 374 articles other than the full-length original articles were excluded directly from the total search contents during the screening step. Forty-seven studies proceeded to the eligibility assessment step. Due to study duplication, 24 studies were excluded, and the remaining 23 were evaluated carefully to examine whether they matched our inclusion criteria. After rigorous evaluation and validation, 13 studies were excluded due to study irrelevance. A total of 10 studies were decided to be included in the systematic review and meta-analysis as they matched our inclusion criteria properly (Fig. 1).

### 3.2. Quality assessment of the included studies

Nine different questions were asked to each included studies to assess their quality (Table 1). The questions were answered in Yes (score 1), No (score 0), unclear (score 0), Not reported or NA (no score). The quality of the individual studies was assessed based on the calculation strategy we found that all eleven studies scored more than 80% and thus were high-quality studies. All studies except Remis 2012 and Barbacci 1991 obtained a perfect score of 100.0% although both crossed the percentage range of 80.0 to be called high-quality studies (Table 1).

**Table 1:** Quality assessment of the included studies.

Study ID	1	2	3	4	5	6	7	8	9	Overall score (%)
Romero-Gutiérrez (2009)^[30]^	Y	Y	Y	Y	Y	Y	Y	Y	Y	100.0
Viani (2003)^[31]^	Y	Y	Y	Y	Y	Y	Y	NR	Y	100.0
Ratnam (1996)^[32]^	Y	Y	Y	Y	Y	Y	Y	Y	Y	100.0
Remis (2012)^[33]^	Y	Y	Y	Y	N	Y	Y	NR	Y	87.5
Barbacci (1991)^[34]^	Y	Y	N	Y	Y	Y	Y	Y	Y	88.9
Viani (2013)^[35]^	Y	Y	Y	Y	Y	Y	Y	NR	Y	100.0
Lee (2011)^[17]^	Y	Y	Y	Y	Y	Y	Y	Y	Y	100.0
Jamieson (2007)^[36]^	Y	Y	Y	Y	Y	Y	Y	NR	Y	100.0
Viani (2016)^[37]^	Y	Y	Y	Y	Y	Y	Y	NR	Y	100.0
Carvalho (2022)^[38]^	Y	Y	Y	Y	Y	Y	Y	NR	Y	100.0

Here, 1. Was the research question appropriate? 2. Is the target/study population clearly defined? 3. Were any inclusion and/or exclusion criteria mentioned? 4. Was any time frame mentioned? 5. Are nonresponders clearly described? 6. Is the sample representative of the target population? 7. Were data collection methods standardized? 8. Was the HIV measuring kit/tool validated? 9. Did the authors use statistical analyses?

N = No, NA = not applicable, NR = not reported, U = unclear, Y = yes.

### 3.3. Major characteristics of the included studies

Among the included studies, 4 were conducted in Mexico, 4 in Canada and the remaining 2 in USA. The studies were conducted in various hospitals, health institutions and public health laboratories across different states and cities of Mexico, Canada and USA within the years 1991 and 2022, each with a distinct study duration. The included studies were of 3 types including 6 cross-sectional studies, 2 cohort studies and 2 observational cohort studies. All the included studies were conducted in a single state or city except the MIRIAD study described by Jamieson et al which was conducted across 6 different states (Atlanta, Baton Rouge, Chicago, Miami, New Orleans, and New York) in USA^[36]^ (Table 2).

**Table 2:** Major characteristics of the included studies.

Study ID	Location	Study type	Duration of the study	Study setting	Participants demographics				HIV detection test	HIV test kit/method	HIV confirmation test kit/method	References
					Age	HIV cases (event)	No. of pregnant women (total)	HIV %				
Romero-Gutiérrez (2009)	Leon, Mexico	Cross-sectional study	December 18, 2003, to February 28, 2005	Hospital of Obstetrics and Gynecology of the Mexican Institute of Social Security, Leon, Mexico	25.6 ± 0.1	2	2257	0.089	Serum Antibody test	Double ELISA (Abbott Axsym System, Wiesbaden, Germany)	Western Blot assay (New Lav-Blot I, Bio-Rad Laboratories, Marnes, France)	^[30]^
Viani (2003)	California, Mexico	Cross-sectional study	January 1998 to December 2000	Tijuana General Hospital	NR	108	19,825	0.545	Serum Antibody test	Rapid HIV serological test	NR	^[31]^
Ratnam (1996)	Newfoundland, Canada	Cross-sectional study	November 1, 1991, to October 31, 1992	Newfoundland Provincial Public Health Laboratory, St. John’s	15 to 44	13	14,911	0.087	Serum Antibody test	EIA kit (HIVAB HIV-1 EIA, Abbott Laboratories, North Chicago, Ill.)	Western blot technique	^[32]^
Remis (2012)	Ontario, Canada	Cohort study	January 1999 to December 2010	The Toronto Public Health Department,	<20 to 40+	74	281,961	0.026	Serum Antibody test	EIA	Western blot technique	^[33]^
Barbacci (1991)	Baltimore, USA	Observational cohort study	February, 1987 to May, 1989	The obstetrics clinic of the Johns Hopkins Hospital	23	80	1961	4.080	Serum Antibody test	ELISA (Genetics Systems, Seattle, WA, or Organon-Teknika, Charleston, SC)	Western blot (Du Pont)	^[34]^
Viani (2013)	Baja California, Mexico	Cohort study	September 2007 to July 2008	Tijuana General Hospital	23.5	24	3375	0.711	Serum Antibody test	EIA, HIV-1/2 (Abbott Diagnostics, North Chicago, IL), ELISA	Western blot	^[35]^
Lee (2011)	Alberta, Canada	Cross-sectional	April 30, 2007 to November 23, 2009	Five acute care hospitals from urban and rural regions, Alberta Provincial Public Health Laboratory	24–51	24	1737	1.382	Serum Antibody test	INSTI^TM^ HIV-1/HIV-2 Rapid Antibody Test (bioLytical^TM^ Laboratories, Richmond, BC, Canada)	Western blot (Genetic Systems^TM^ HIV-1 Western Blot, Bio-Rad Laboratories, Montreal, QC, Canada)	^[17]^
Jamieson (2007)	Atlanta, Baton Rouge, Chicago, Miami, New Orleans, and New York USA	Cross-sectional	November, 2001 to February, 2005	MIRIAD Hospital, Kampala, Uganda (personal communication, M.G. Fowler) and St. Petersburg, Russia,15 and 15 other hospitals	20–35	52	7753	0.671	Serum Antibody test	EIA Abbott HIV-1/HIV-2 EIA (Abbott Laboratories, Abbott Park, IL), HIV-1/HIV-2 peptide EIA (BioRad Laboratories, Hercules, CA), and 3 used the bioMerieux Vironostika HIV-1 ELISA kit (bioMerieux, Durham, NC)	Western blot	^[36]^
Viani (2016)	Tijuana, Mexico	Cross-sectional study	Between 2007 and 2008	Tijuana General Hospital, Mexico.	Migrants (22.7 vs 23.4), local (16.7 vs 17.2)	21	3331	0.630	Serum Antibody test	Rapid HIV test	Western blot	^[37]^
Carvalho (2022)	Canada	Observational cohort study	2003 to 2020.	NR	NR	40	2720	1.471	Serum Antibody test	NR	NR	^[38]^

EIA = enzyme immunoassay, ELISA = enzyme-linked immunosorbent assay, HIV = human immunodeficiency virus, NR = not reported.

Twenty-eight thousand seven hundred eighty-eight participants of 4 studies conducted in Mexico,^[30,31,35,37]^ 301,329 of 4 studies conducted in Canada,^[17,32,33,38]^ and 9714 participants of 2 studies in USA^[34,36]^ with a total of 339,831 participants with varying age ranges were reported in this systematic review and meta-analysis. Participants’ age range was not reported in 2 studies with study IDs of Viani 2003 and Carvalho 2022 conducted by Viani et al and Carvalho et al, respectively.^[31,38]^

For primary detection of HIV all the 10 studies relied on serum antibody test but applied a wide varieties of HIV test methods/kits, for example, double enzyme-linked immunosorbent assays (ELISA) tests (Abbott Axsym System, Wiesbaden, Germany), rapid HIV serological test, enzyme immunoassay (EIA) kit (HIVAB HIV-1 EIA, Abbott Laboratories, North Chicago, IL), ELISA (Genetics Systems, Seattle, WA, or Organon-Teknika, Charleston, SC), EIA, HIV-1/2 (Abbott Diagnostics, North Chicago, IL), confirmatory enzyme immunoassay (Abbott Diagnostics, North Chicago, IL), INSTITM HIV-1/HIV-2 Rapid Antibody Test (bioLyticalTM Laboratories, Richmond, BC, Canada), EIA Abbott HIV-1/HIV-2 EIA (Abbott Laboratories, Abbott Park, IL), HIV-1/HIV-2 peptide EIA (BioRad Laboratories, Hercules, CA), and bioMerieux Vironostika HIV-1 ELISA kit (bioMerieux, Durham, NC) (Table 2). Western blot technique was used as the HIV confirmation test method in all the studies except Viani 2003 and Carvalho 2022 in which no HIV confirmation test method was reported^[31,38]^ (Table 2).

### 3.4. Meta-analysis

**Figure 2.**
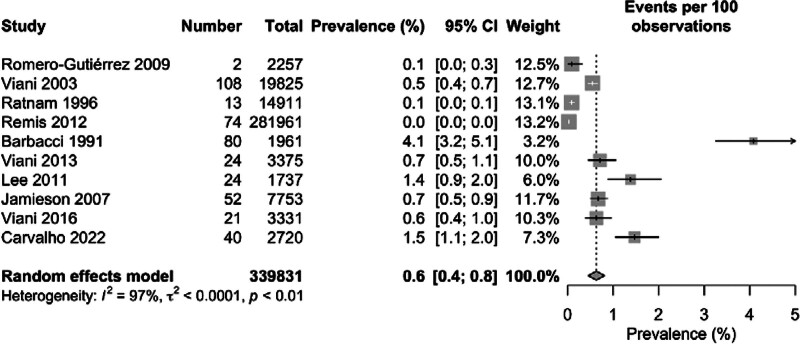
Geographical presentation of the prevalence of nephropathy among diabetes patients in the North American region.

Among the 339,831 overall pregnant women included in this meta-analysis, 438 were diagnosed as HIV positive. The overall pooled prevalence of HIV among pregnant women was 0.6% with 95% CI of 0.4 to 0.8 (*I*^2^ = 97%). The highest prevalence rate of 4.1% (95% CI: 3.2–5.1) was found in Barbacci 1991 whereas zero prevalence rate (95% CI: 0.00) was found in Remis 2012. Notably low prevalence of 0.1% (95% CI: 0.0–0.1, 0.0–0.3) was reported in both Ratnam 1996 and Romero-Gutiérrez 2009 conducted in Canada and Mexico respectively. Several other studies, for example, Viani 2003 (0.5% [95% CI: 0.4–0.7]), Viani 2016 (0.6% [95% CI: 0.4–1.0]), Jamieson 2007 (0.7% [95% CI: 0.5–0.9]) and Viani 2013 (0.7% [95% CI: 0.5–1.1]) showed lower prevalence rates as compared to Lee 2011 (1.4% [95% CI: 0.9–2.0]) and Carvalho 2022 (1.5% [95% CI: 1.1–2.0]). A significantly high degree of heterogeneity (*I*^2^ = 97%) was found among the included studies in this meta-analysis (Fig. 2).

**Figure 3.**
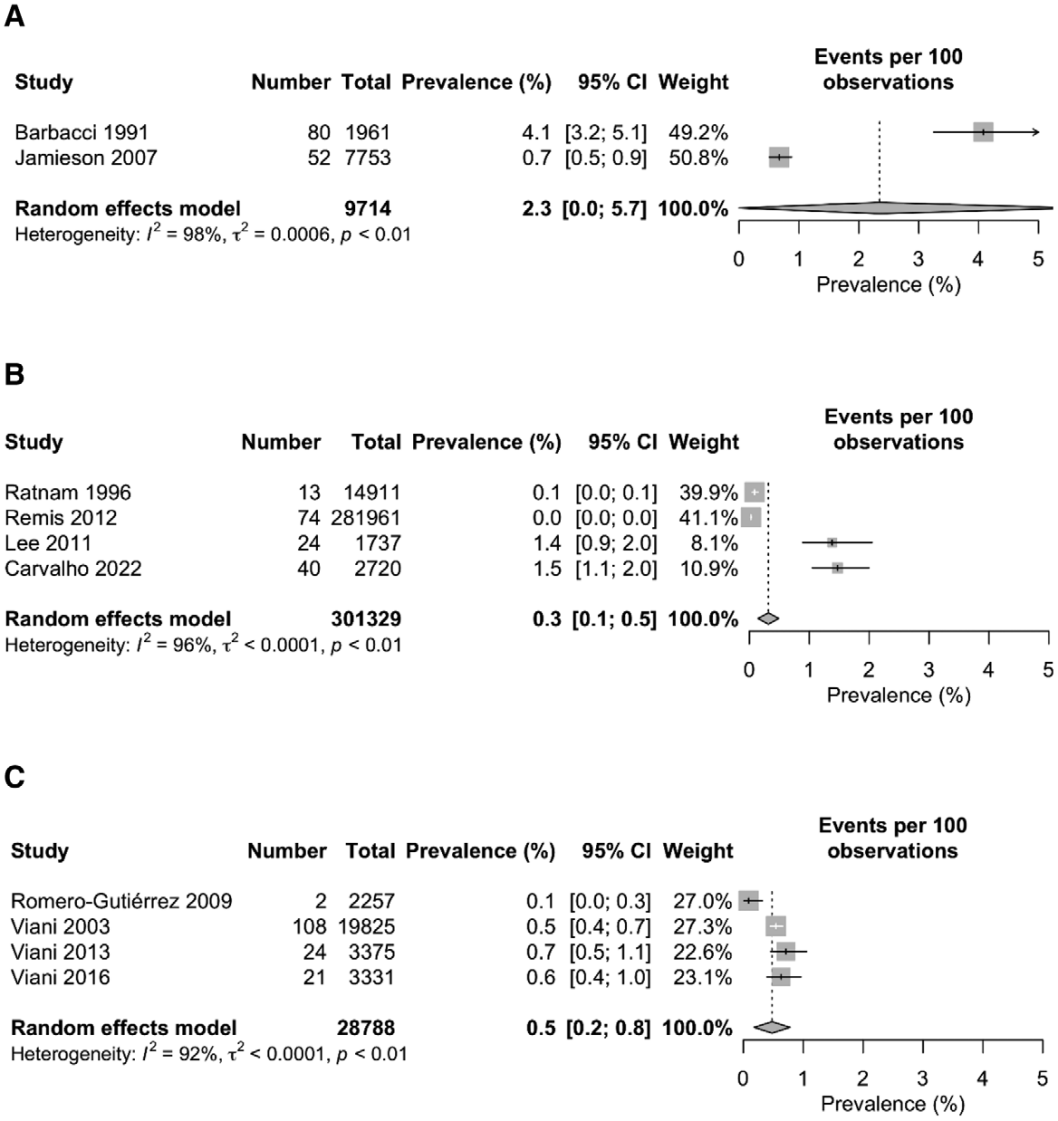
Forest plot of the pooled prevalence of nephropathy among diabetes patients.

**Figure 4.**
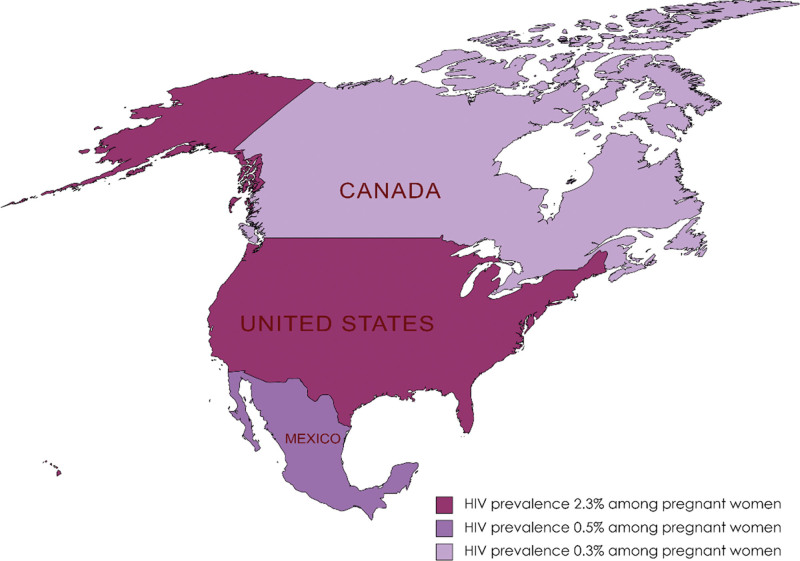
Forest plot of the prevalence of nephropathy among diabetes patients in (A) Canada, (B) USA, and (C) Mexico.

The pooled prevalence of HIV among pregnant women in individual countries varied from each other with the lowest prevalence of 0.3% (95% CI: 0.1–0.5) in Canada, 0.5% (95% CI: 0.2–0.8) in Mexico and 2.3% (95% CI: 0.0–5.7) in the USA. The *I*^2^ statistics showed a high degree of heterogeneity among the studies conducted in all 3 countries ranging from 92% among Mexican studies to 98% among the USA studies (Fig. 3). The geographical map of the North American region represents the different prevalence area of HIV among the pregnant women (Fig. 4).

### 3.5. Possible sources of heterogeneity

Separately, studies of the USA were found to have the highest heterogeneity (*I*^2^ = 98%), followed by heterogeneity of the studies of Canada (*I*^2^ = 96%) and Mexico (*I*^2^ = 92%). Therefore, it indicates that the studies of the USA (i.e., Barbacci 1991 and Jamieson 2007) were the major sources of heterogeneity, followed by the studies of Canada (i.e., Ratnam 1996, Remis 2012, Lee 2011, and Carvalho 2022) and Mexico (i.e., Romero-Gutiérrez 2009, Viani 2003, Viani 2013, and Viani 2016) (Fig. 3).

### 3.6. Publication bias and sensitivity analysis

**Figure 5.**
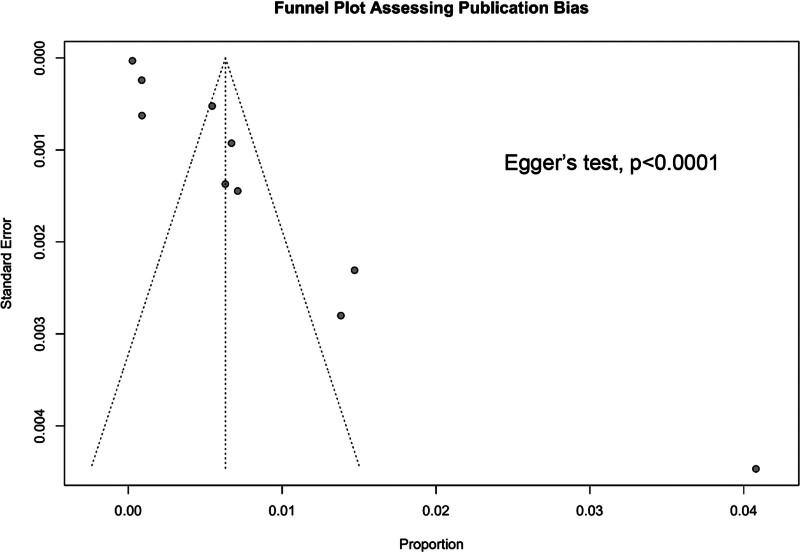
Funnel plot assessing publication bias.

**Figure 6.**
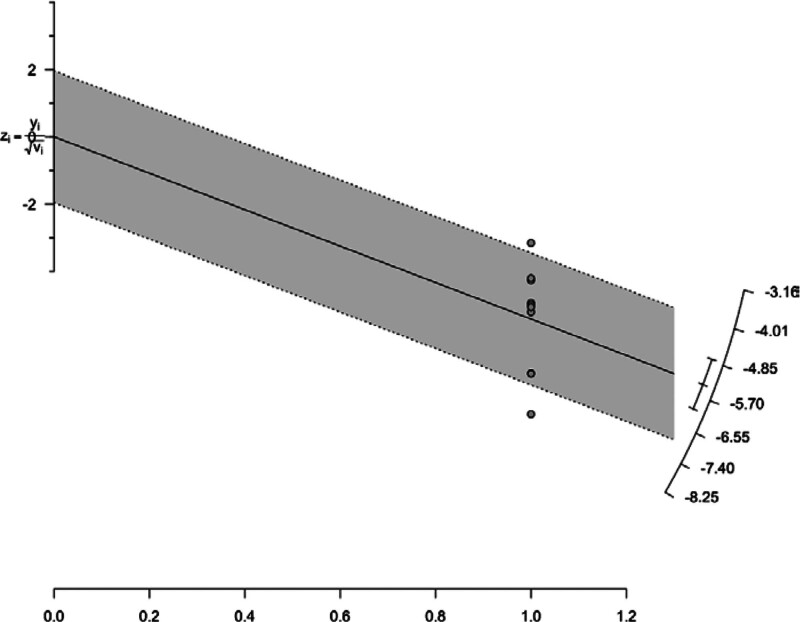
Galbraith plot indicating outlier studies.

**Figure 7.**
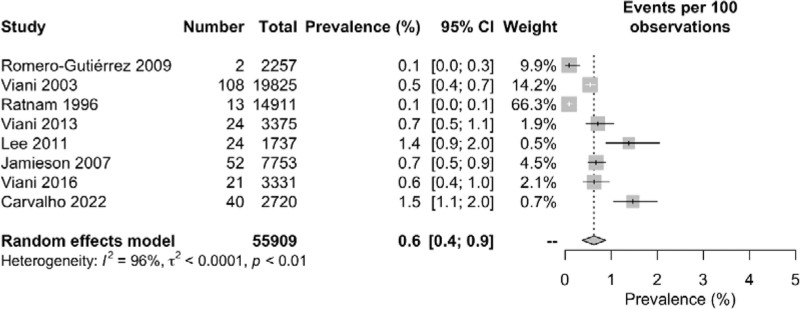
Forest plot of the prevalence of nephropathy among diabetes patients using random effect model (excluding outlier studies).

Initially, the plausible sources of publication bias were visualized through a funnel plot with Egger test, where the publication bias was found to be highly significant (*P* < .0001) (Fig. 5). For further identification and confirmation, a Galbraith plot was constructed which detected Barbacci 1991 and Remis 2012 outliers (Fig. 6). To determine the impact of the outlier study on the overall effect size, Barbacci 1991 and Remis 2012 were excluded from the analysis and a forest plot was reconstructed using random effects model which showed the overall pooled prevalence of 0.6% (95% CI: 0.4–0.9) same as the previous forest plot with the outlier study indicating the strength of our primary analysis. *I*^2^ statistics showed slightly decreased heterogeneity of 96% after excluding the outlier studies from the analysis (Fig. 7).

**Figure 8.**
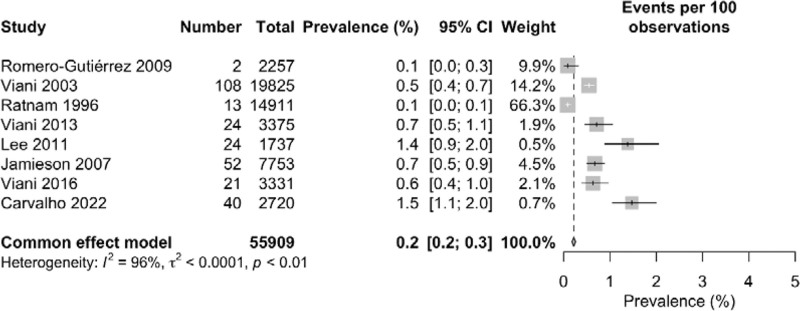
Forest plot of the prevalence of nephropathy among diabetes patients using fixed effect model (excluding outlier studies).

When a forest plot was reconstructed without Barbacci 1991 and Remis 2012 using fixed effects model, the pooled prevalence dropped significantly from 0.6% (95% CI: 0.4–0.9) in random effects model to 0.2% (95% CI: 0.2–0.3). The *I*^2^ statistics showed 96% heterogeneity which is similar to the heterogeneity percentage found using random effects model (Fig. 8).

## 4. Discussion

In this systematic review and meta-analysis, the prevalence of human immunodeficiency virus among pregnant women in 3 major North American countries: the USA, Canada, and Mexico were analyzed and discussed. To the authors’ knowledge, this is the first meta-analysis conducted on the HIV prevalence among pregnant women in the North American region. We found an overall pooled prevalence of 0.6% with a 95% CI of 0.4 to 0.8. In Canada and Mexico, the prevalence are 0.3% and 0.5% respectively whereas in the USA, the prevalence rate is 2.3% which was significantly higher as compared to the Canada and Mexico (Fig. 3).

Currently, 1.2 million people in the USA are living with HIV. The latest Centers for Disease Control and Prevention report also suggests that 13% of HIV patients are unaware of their condition and testing is required.^[39]^ In the USA, 34,800 new cases of HIV were reported in 2015 which after a period of general stability decreased by 8% from 37,800 in 2015 to 34,800 in 2019. In Canada, a total of 2122 HIV cases were reported in 2019 which is only 5.6 per 100,000 population.^[40]^ However, there were roughly 14,200 reported HIV cases in Mexico in 2021. During that year, the states of Mexico, Veracruz, and Jalisco had the highest HIV diagnosis rates, each surpassing a 1000 patients.^[41]^

On the other hand, it was found that >2.3 million people in the WHO European Region, particularly in the Eastern part of the region are infected with HIV. It was reported that nearly 107,000 people were diagnosed with HIV in the European Region in 2022, including around 17,000 in the EU/EEA. Among the 53 countries in the WHO European region, a total of 142,197 newly diagnosed HIV cases were reported in 50 countries reaching an overall count of 1,840,136 since 1980. Eastern Europe holds the highest infection rate with 43.2 per 100,000 persons constituting of 77% of total HIV diagnosed cases in 2014.^[42]^ Africa, the hotspot of HIV infection reported of having 25.7 million HIV-infected persons out of total 37.9 million HIV-infected persons globally with 1.1 million newly diagnosed cases alone in 2018.^[43]^

Particularly the African: Sub-Saharan Africa is the epicenter of the HIV pandemic, with 25.5 million people living with HIV. High age-standardized prevalence, death, and disability-adjusted life years are predominant in Africa, especially in the Southern Sub-Saharan region.^[44,45]^ At Nigeria region, Eaton 2014 estimated incidence rates ranging from 2.4% in the North-Central zone to 25.42% in the South-South, totaled at 7.22%.^[46]^ The prevalence of HIV infection in South America is also higher than that of North American countries. According to the report of the Joint United Nations Program on HIV/AIDS, a total of 1,900,000 adults and children were living with HIV in Latin American and Caribbean regions with an overall prevalence of 0.5% in 2018.^[47]^

The HIV prevalence rate is directly or indirectly influenced by several factors such as awareness among the population, knowledge, and attitudes of individuals, healthcare structure and system, HIV testing accessibilities, education level, knowledge regarding HIV pathogenesis and prevention, etc. These factors correspond to one another. For example, if the population is very large it may be difficult to create awareness simultaneously and the progression may be slow while also it will be difficult to test so many participants in a certain period of time making it very hard to keep tabs on HIV spreading criteria.^[48–51]^ The healthcare system in the United States is comparatively well-developed, enabling earlier testing and diagnosis of individuals and prompt treatment and care. Access to medical services, such as HIV testing, is available to everyone. Availability of Medications such as ART is widely available in the USA. HIV education and awareness initiatives have been widely implemented among the public. The U.S. has implemented needle exchange programs to reduce the risk of HIV transmission.^[52]^

Among the included studies Barbacci 1991 estimated the higher prevalence rate in the Baltimore, USA region and reported that out of 1961 pregnant women 80 were HIV positive in a survey conducted in 1991. At the time, there was a lack of awareness and precaution among the general population along with health care system that was not advanced. Moreover, it was not possible to screen people early, diagnose them, or provide them with appropriate medication which might be responsible for such a high prevalence of HIV in USA.^[34]^

Lee2011 and Carvalho 2022 estimated a moderately higher prevalence in the Canada region but much lower than Barbacci 1991. Lee 2011 survey reported 24 HIV-positive cases out of 1737 pregnant women whereas the survey of Carvalho 2022 reported 40 positive cases out of 2720 pregnant women.^[17,34,38]^ One of the primary causes of the higher incidence is the USA’s denser population than Canada’s. HIV prevalence has started to decline over time.

Again higher degree of heterogeneity was found in both the overall (97%) as well as the country-wise prevalence analysis (>90%) which indicates substantial variations among the included studies making it difficult to generalize the meta-analysis outcomes to the entire study populations of the 3 countries.^[53,54]^ These kinds of variations may result from several factors including variation in study types, study duration and study setting, sample size, detection and confirmation tests, types of kits or methods, etc (Table 2).

Not only the 3 countries included in our meta-analysis, HIV prevalence rate among pregnant women across different countries in North America is relatively low.^[55]^ Common factors that reinforce the reduced number of prevalence rates in North America are based on their way of life and infrastructure of society. Some basic factors including effective HIV prevention and education initiatives launched throughout North America, assisting to spread knowledge about the disease and encouraging healthy sex behaviors. These initiatives have focused on at-risk groups such sex workers, intravenous drug users, and men who have sex with males. Other implementations such as, HIV testing access, needle exchange programs, effective law enforcement, prevention treatments, and non-transmittable. Also, their sexual behavior is within the threshold point that causes a breakout of transmission.^[55]^ Interestingly, these types of initiatives and HIV prevention activities are rare in African regions specially the Sub-Saharan Africa which is called the epicenter of HIV pandemic. HIV epidemics in this region are mainly driven by heterosexual transmission and people are largely rural and nomadic.^[45]^ For instances, WHO claimed a 41% prevalence rate of HIV in Nigeria, which is 6 times higher than the combined estimate obtained.^[56]^

Studies estimated that the prevalence of HIV among expectant Nigerian women is higher than that of other developing nations like Brazil (0.38%), Ethiopia (5.74%), and Tanzania (5.6%).^[57–59]^

A number of reasons act as indirect confessions to why HIV is so severe in Africa. Some sources suggest that African HIV cases arise from cultural activities solely on the belief that they are being punished by God and thus lack of education creates a situation in which they are not taking proper steps to do any prevention. Illiteracy and lack of awareness is the key role playing here.^[60]^ Another burning reason within their community is poverty. Due to poverty there have been many prostitution activities, migration, teenage marriages which impact the HIV spread condition to an alarming rate due to rapid transmission frequency. Therefore, their society plunges into the clutches of HIV. Different African regions practice polygamy and promiscuity, which helps HIV/AIDS spread. Other reasons also include sexual violence against women, stigma, and discrimination.^[61,62]^

Geopolitical reasons can also indirectly influence the spread of HIV. Some of the vital reasons are the conflict and political Instability, Economic Sanctions and Geopolitical alliances.

Territories suffering conflict and political unrest frequently have interrupted healthcare systems, debilitated infrastructure, and sizable populations that have been displaced. Due to disruptions in healthcare, population migration, and other variables, these situations can make it more difficult to provide HIV prevention and treatment services and raise the risk of HIV transmission. This can lead to HIV spread on mothers and their children due to lack of care and attention and as a result HIV testing will be reduced in that unstable region.

Countries that have been subjected to economic sanctions may find it more difficult to get basic medical supplies and medications, such as the antiretroviral drugs needed to treat HIV/AIDS. This may result in scarcities and restricted access to HIV therapy for those who need it. It will cause negligence to women with HIV and they would not receive enough treatment.

Geopolitical alliances and conflicts can have an impact on how international aid and collaboration for HIV/AIDS initiatives is distributed. Political factors may have an impact on the funding and support for preventive and treatment initiatives, which may change the resources that are available to impacted nations. Geopolitical factors can have an impact on diplomatic relations and talks regarding global health crises, such as HIV/AIDS. The outcome of these discussions may have an impact on the willingness of nations to cooperate and pool their expertise and resources in the battle against the pandemic. These factors can indirectly cause lack in care and treatment plan for HIV pregnant women.^[63]^ These factors define the reason behind the variation of the prevalence rate among other countries or African continent with North American region.

Although the current systematic review and meta-analysis did not cover the entire North America, it represented the scenario of a major region of this continent which can be utilized to perform future research regarding the prevalence of HIV among pregnant women in other parts of this continent. A well-developed healthcare system, HIV prevention programs, the availability of medications, public health education and awareness campaigns about HIV, maintaining precautions during needle exchange, and other actions can reduce HIV prevalence to almost zero.^[64]^ Indeed, proper designing and implementation of preventive measures to reduce the prevalence of HIV, especially among pregnant women is undoubtedly a great challenge to the healthcare system and scientific community throughout the world. Nevertheless, careful planning and preventive execution can reduce the HIV population significantly which has been reflected in the case of the North American region in this study.

## 5. Conclusion

This systematic review and meta-analysis indicated that the prevalence of HIV infection among pregnant women in the USA, Canada, and Mexico was notably low. Although the evidence of quite high prevalence in the USA was found in a study of Barbacci et al in 1991, the prevalence rate decreased significantly over time possibly due to progress in public awareness and knowledge about HIV infection, transmission and prevention, advanced healthcare facilities and HIV detection methods, HIV testing accessibilities, and better socio-economic conditions. Further improvement of public awareness, knowledge, attitude, and practices regarding HIV prevention as well as more HIV prevention campaigns and advanced healthcare structures are required to reduce the HIV prevalence among pregnant women to zero.

## 6. Limitations of the study

This study only included original peer-reviewed research articles from 3 different databases for the systematic review and meta-analysis part. No data from any websites were obtained.

## Author contributions

**Conceptualization:** Nosheena Akhter Shabbir, Alif Hasan Pranto, Hasan Shahriyer Tonmoy, Ehsan Suez.

**Data curation:** Aiza Ali Akbar, Syeda Wajiha Batool, Amna Akbar, Muhammad Iftikhar Khattak, Attarab Shakeel.

**Formal analysis:** Sara Bashir Kant, Md Rashedul Islam, M. M. Rubaiyat Muntasir Meem, Shahad Saif Khandker, Amna Akbar.

**Methodology:** Sara Bashir Kant, Kainat Rashid, Uzma Hafeez, Jemema Zaman, Dewan Zubaer Islam, Ehsan Suez, Amna Akbar, Sarosh Khan Jadoon.

**Resources:** Uzma Hafeez, Jemema Zaman, M. M. Rubaiyat Muntasir Meem, Shahad Saif Khandker, Muhammad Iftikhar Khattak.

**Software:** Shahad Saif Khandker, Sarosh Alvi.

**Supervision:** Nosheena Akhter Shabbir, Alif Hasan Pranto, Amir Iqbal Ali.

**Writing – original draft:** Kainat Rashid, Hasan Shahriyer Tonmoy, Md Rashedul Islam, Dewan Zubaer Islam.

**Writing – review & editing:** Aiza Ali Akbar, Syeda Wajiha Batool, Amir Iqbal Ali, Sarosh Khan Jadoon, Attarab Shakeel, Maryam Zubair, Sarosh Alvi.

## Supplementary Material


