# Supplementary material for: Prevalence of HIV/AIDS among pregnant women in North American region: A systematic review and meta-analysis
Source: Medicine (Baltimore). 2024 Nov 1;103(44):e40339. doi: 10.1097/MD.0000000000040339 (PMC11537646; doi:10.1097/MD.0000000000040339)
Supplement: Supplementary file 1 [file medi-103-e40339-s001.docx]

Supplementary table 1: Search Strategy

| **Databases** | **Search Strategy** | **Result** |
| --- | --- | --- |
| **PubMed** | ((((((Human immunodeficiency virus[Title/Abstract]) OR (HIV[Title/Abstract])) AND (((((prevalen*[Title/Abstract]) OR (inciden*[Title/Abstract])) OR (epidemiolog*[Title/Abstract])) OR (frequen*[Title/Abstract])) OR (occurren*[Title/Abstract]))) AND (((((pregnan*[Title/Abstract]) OR (prenatal[Title/Abstract])) OR (antenatal[Title/Abstract])) OR (perinatal[Title/Abstract])) OR (maternal[Title/Abstract])))))) AND (((Canada[Title/Abstract]) OR (Mexico[Title/Abstract])) OR (USA[Title/Abstract])) | 1**81** |
| **Google Scholar** | allintitle: pregnant HIV USA  allintitle: pregnancy HIV USA  allintitle: pregnant AIDS USA  allintitle: pregnancy AIDS USA  allintitle: pregnant Human immunodeficiency USA  allintitle: pregnancy Human immunodeficiency USA  allintitle: pregnant HIV Canada  allintitle: pregnancy HIV Canada  allintitle: pregnant AIDS Canada  allintitle: pregnancy AIDS Canada  allintitle: pregnant Human immunodeficiency Canada  allintitle: pregnancy Human immunodeficiency Canada  allintitle: pregnant HIV Mexico  allintitle: pregnancy HIV Mexico  allintitle: pregnant AIDS Mexico  allintitle: pregnancy AIDS Mexico  allintitle: pregnant Human immunodeficiency Mexico allintitle: pregnancy Human immunodeficiency Mexico | **128** |
| **ScienceDirect** | Title, abstract, keywords: pregnant HIV USA  Title, abstract, keywords: pregnancy HIV USA  Title, abstract, keywords: pregnant AIDS USA  Title, abstract, keywords: pregnancy AIDS USA  Title, abstract, keywords: pregnant HIV Canada  Title, abstract, keywords: pregnancy HIV Canada  Title, abstract, keywords: pregnant AIDS Canada  Title, abstract, keywords: pregnancy AIDS Canada  Title, abstract, keywords: pregnant HIV Mexico  Title, abstract, keywords: pregnancy HIV Mexico  Title, abstract, keywords: pregnant AIDS Mexico  Title, abstract, keywords: pregnancy AIDS Mexico | **112** |
